# Supplementary material for: Revealing low-loss dielectric near-field modes of hexagonal boron nitride by photoemission electron microscopy
Source: Nat Commun. 2023 Aug 10;14:4837. doi: 10.1038/s41467-023-40603-4 (PMC10415285; doi:10.1038/s41467-023-40603-4)
Supplement: Supplementary file 2 — Supplementary Information [file 41467_2023_40603_MOESM2_ESM.pdf]

Supplementary Information for

Revealing low-loss dielectric near-field modes of hexagonal boron nitride by photoemission electron microscopy

Yaolong Li, Pengzuo Jiang, Xiaying Lyu, Xiaofang Li, Huixin Qi, Zhaohang Xue, Jinglin Tang, Hong Yang, Guowei Lu, Quan Sun*, Xiaoyong Hu*, Yunan Gao*, Qihuang Gong

Corresponding author: Email: sunquan@ydioe.pku.edu.cn (Q.S.); xiaoyonghu@pku.edu.cn (X.H.); gyn@pku.edu.cn (Y.G.)

Supplementary Note 1
<u>Sample characterizations</u>

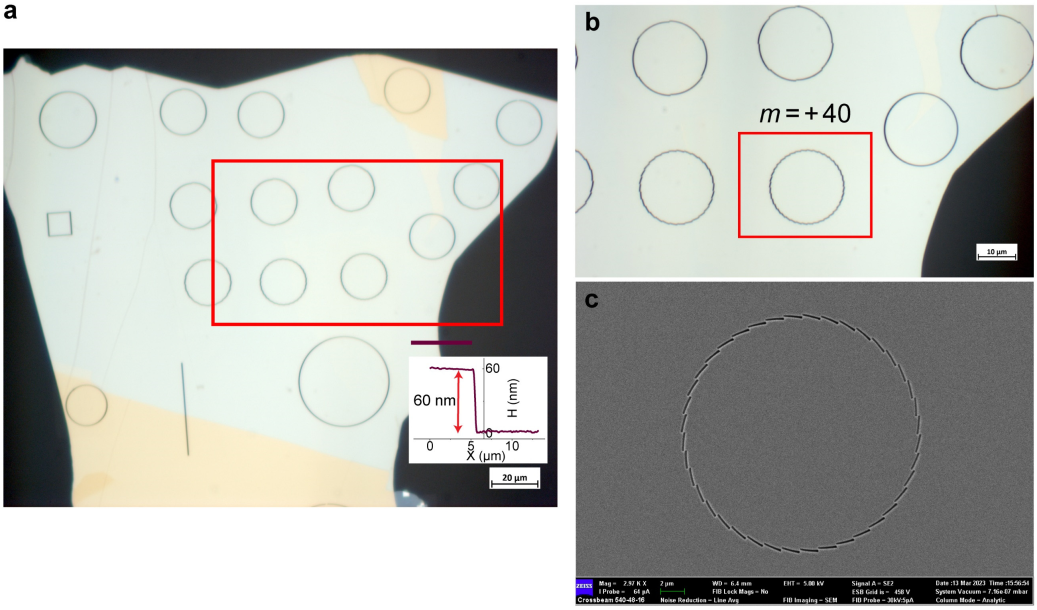

Supplementary Figure 1. Sample characterizations. Optical and SEM images of typical hBN samples, SEM image in (**c**) corresponds to the sample in Fig. 1c of the main manuscript.

Supplementary Note 2
<u>Laser pulse characterizations</u>

The characterizations of the SHG pulses are shown in Supplementary Figure 2.

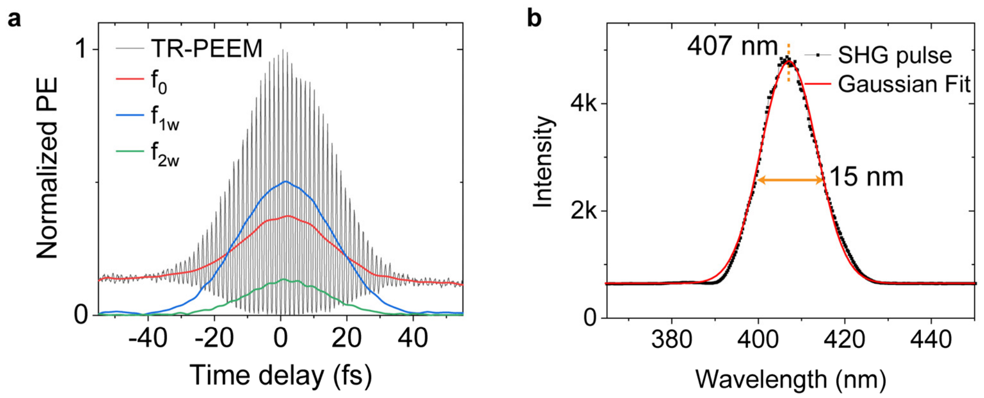

Supplementary Figure 2. Characterizations of the ultrashort pulses for TR-PEEM measurements. (**a**) TR-PEEM signals on hBN for the characterization of the SHG pulses, the FWHM (full width at half maximum) of f_0 is about 30.6 fs, thus the pulse duration is calculated to be $30.6/\sqrt{2} = 21.6$ fs. (**b**) Spectrum of the SHG pulses with a central wavelength of 407 nm and spectral width of 15 nm.

Supplementary Note 3

<u>Discussion on the selective excitation of TE modes in thin planar waveguide</u>

Here, we will demonstrate that when the hBN waveguide is thin enough (e.g. <100 nm), only the TE mode can be efficiently excited whereas the TM mode is negligible, so that we can use the TE mode of the waveguide to construct dielectric near-field modes. The discussions on the intensity of TE and TM modes with different hBN thicknesses are as follows.

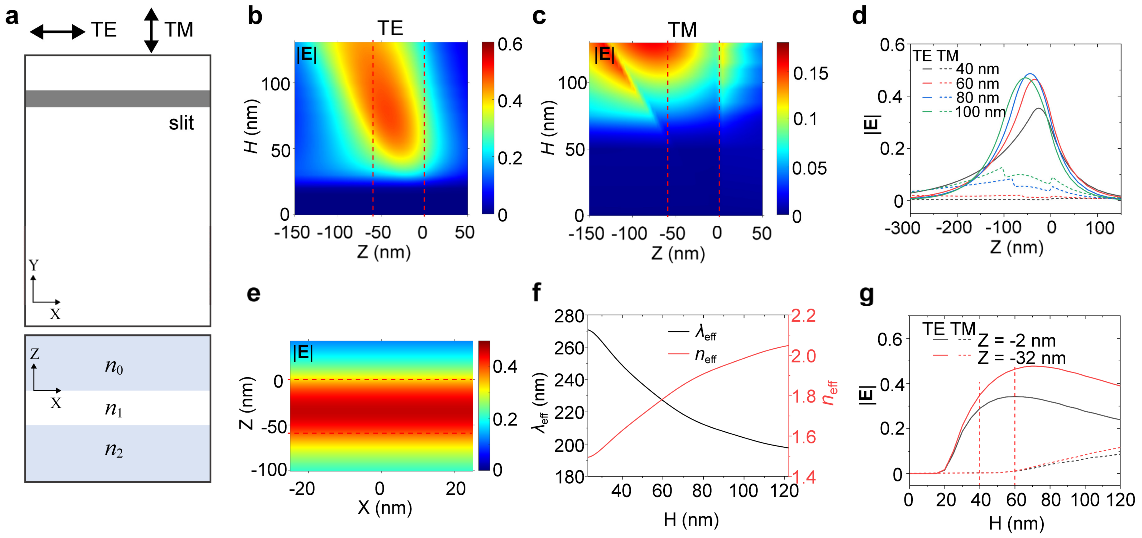

Supplementary Figure 3. Excitation of TE and TM modes in the thin planar waveguide. (**a**) Layout of the planar waveguide with a slit as the coupler, where Z=0 is set at vacuum/hBN interface. (**b**) Electric field amplitude |**E**| along Z axis for TE mode excited with polarization parallel to the slit with different

thicknesses (H) of hBN layer. (**c**) |**E**| along Z axis for TM mode excited with polarization vertical to the slit. (**d**) Crosscut lines from (b,c) with hBN thickness 40–100 nm, indicating that the TM mode is much weaker than TE mode. (**e**) Typical mode profile of TE mode with a thickness of 60 nm. (**f**) Effective refractive index n_{eff} and the corresponding effective wavelength λ_{eff} of TE mode varying with hBN thickness. (**g**) |**E**| of TE and TM modes versus the thickness of hBN. |**E**| is normalized to the amplitude |$\mathbf{E}_0$| of incident light.

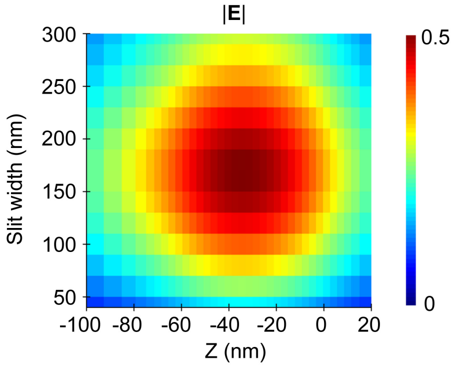

Supplementary Figure 4. Optimization of slit width. Optimization of slit width by the simulation of the amplitude of |**E**| coupled into 60 nm hBN waveguide vs slit width for 410 nm laser. The optimal width is around 180 nm.

The planar waveguide with a slit as the coupler is excited with TE or TM polarized plane beam at normal incidence (Supplementary Figure 3a). The results are simulated with the finite-difference time-domain (FDTD) method with the following setting. The minimum mesh is set as 3 nm and the periodic boundary condition is used along the slit direction to simulate an infinitely long slit, and the wavelength of the excitation beam is set as 410 nm. A time apodization at the start is used to filter the influence of the incident beam. The typical fundamental TE mode with the hBN thickness of 60 nm is shown in Supplementary Figure 3e. For the choice of slit width, the optimized slit width is ~180 nm at the excitation wavelength of 410 nm, as shown in Supplementary Figure 4.

The electric field amplitudes (|**E**|) along the vertical crosscut line varying with hBN thickness for TE and TM modes are presented in Supplementary Figure 3b,c. The crosscut lines at the hBN thickness of 40, 60, 80, and 100 nm are shown in Supplementary Figure 3d for better comparison. For the hBN thickness of 60 nm, the electric field amplitudes at the top surface (Z = −2 nm, that is 2 nm below the vacuum/hBN interface) and central plane (Z = −32 nm) of the hBN waveguide are shown in Supplementary Figure 3g. As we can see, the electric field intensity (|$\mathbf{E}$|^2) of TE mode is much stronger than TM mode when the hBN is thin enough. Typically, at the thickness of 40 (or 60) nm, the electric field intensity ratios of TE/TM modes at the top surface and the central plane

are about 7×10^3 (or 1×10^3) and 1.3×10^4 (or 1.5×10^3), respectively. This means that the fundamental TE mode is the dominant mode in a thin hBN waveguide. Based on this finding, we construct the near-field vortex with fundamental TE mode as demonstrated in the main manuscript. The simulated effective refractive index n_{eff} and the corresponding effective wavelength λ_{eff} of TE mode, varying with hBN thickness, are shown in Supplementary Figure 3f, which are used for designing the relative displacement increment of Archimedean spiral slits. At the hBN thickness of 60 nm, for the excitation wavelength of 410 nm, n_{eff} and λ_{eff} are extracted as 1.78 and 230 nm, respectively. As the hBN is a wide-bandgap semiconductor, the waveguide mode can be formed in a broad wavelength range from infrared to deep ultraviolet (UV) by simply tuning the thickness of hBN. The intensity of TE mode can be pronouncedly enhanced by increasing the number of slits, that is using the Bragg grating coupler.

For the circular slits, strong near-field enhancement of the focusing spot is produced by the near-field vortex excited with the circularly polarized beam. The size of the focusing spot achieves 58 nm for $|\mathbf{E}|^4$, apparently beyond the diffraction limit (Supplementary Figure 5a). Here, $|\mathbf{E}|^4$ is used considering the two-photon process of photoemission in PEEM experiments. The near-field enhancement increases with the radius of the circular slit and the number of slits (circular Bragg grating). Noticeably, the near-field amplitude $|\mathbf{E}|$ increases linearly with the radius of the circular slit due to the low loss of dielectric material (Supplementary Figure 5b). The electric field intensity ($|\mathbf{E}|^2$) can be enhanced over 10^3 times compared with the incident beam by using a circular Bragg grating with 10 slits and an initial radius of $30\lambda_{\text{eff}}$ (Supplementary Figure 5c). In contrast, the near-field intensity of SPP at the surface of the metal film is much weaker and decays fast due to the high loss of metal. In addition, the simulation results above are also applicable to the symmetrical waveguide, that is $n_0 = n_2$, which can be realized with free-standing hBN flakes in experiments.

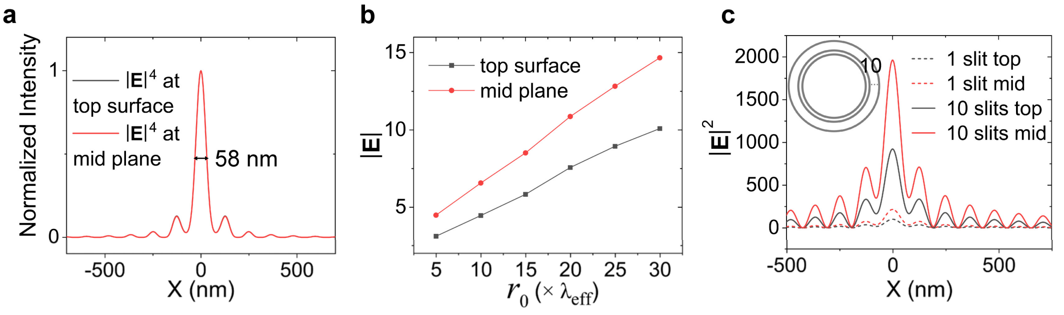

Supplementary Figure 5. Near-field enhancement of the focusing spot. (**a**) Nanofocusing with the lowest vortex excited with a left-handed circularly polarized plane beam (SAM = +1, (E_x = 1, E_y = i)) and the size (FWHM) of the focusing spot for $|\mathbf{E}|^4$ is about 58 nm (~1/7 λ_0 of the incident light). (**b**) Maximum of electric field amplitude ($|\mathbf{E}|$) of the focusing spot varying with the radius of the circular slit at the top surface and central plane, respectively. (**c**) Comparison of $|\mathbf{E}|^2$ with a circular slit and a Bragg grating with 10 slits, the initial radius $r_0 = 30\lambda_{\text{eff}}$. For the grating, the period is 270 nm and the width of the slit is 150 nm.

The selective excitation of TE mode can also be verified with PEEM experiments. As shown in Supplementary Figure 6, the polarization selectivity is observed, indicating the waveguide modes can be excited only when the polarization paralleling to the slit, that is TE mode. In addition, the nanofocusing for left-handed circular and linear polarizations are also presented with the sizes of 65 nm and 60 nm, respectively (Supplementary Figure 6a,b). In addition, the effective wavelength of TE mode can be extracted from the fringe period of the interference between the TE mode and the plane beam at normal incidence. The extracted λ_{eff} and n_{eff} for the hBN thickness of ~60 nm with the excitation wavelength of 410 nm are ~230 nm and ~1.78, respectively, which are consistent with the simulations (Supplementary Figure 6c,d).

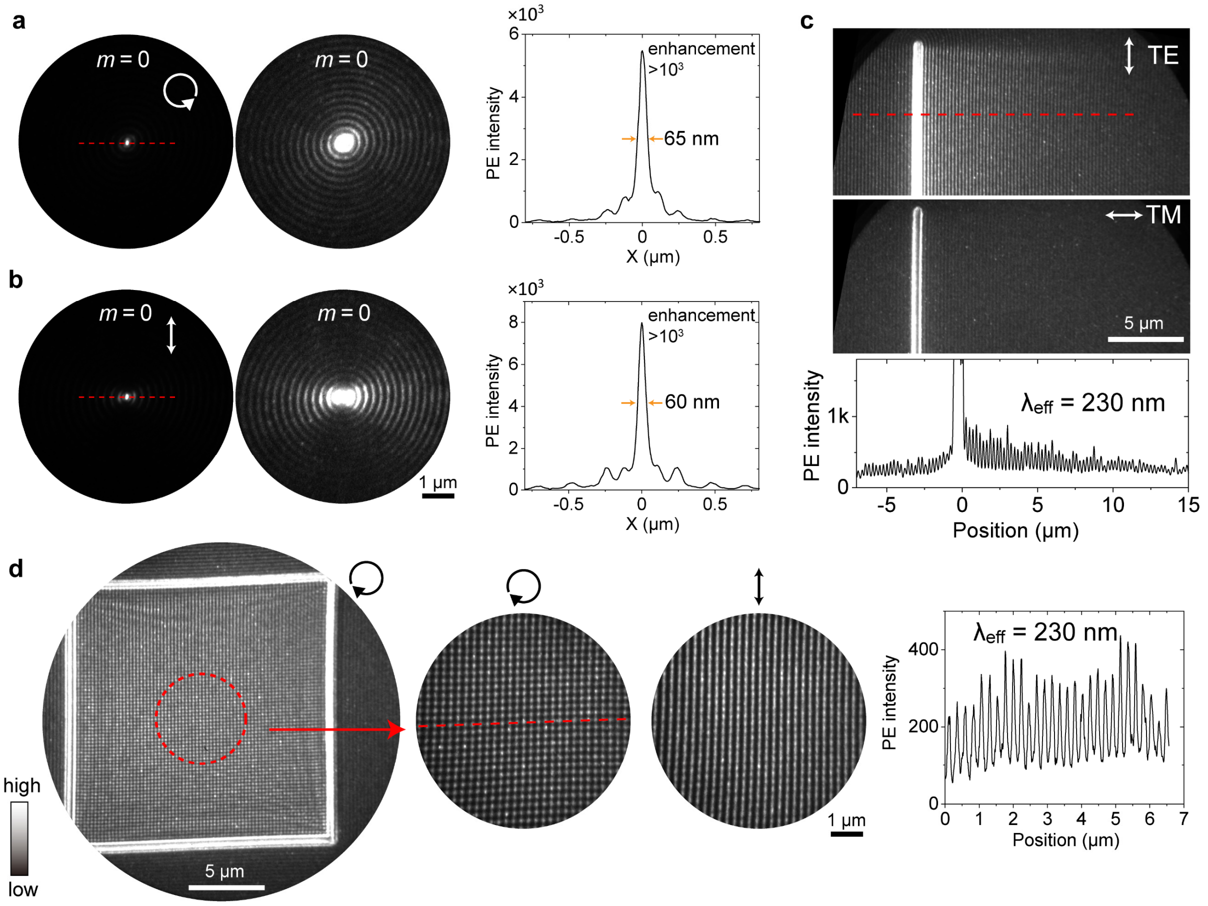

Supplementary Figure 6. Selective excitation of TE mode verified with PEEM experiments. (a,b) PEEM images for ring slit with $m = 0$ excited with left-handed circular and linear polarizations at 410 nm, the focusing sizes are 65 nm and 60 nm, respectively. The left and right images are identical except that the right images are adjusted with brightness and contrast to show the weak interference fringes around the focusing point. (**c**) PEEM images for line slit excited with TE and TM polarizations at 410 nm, the extracted effective wavelength λ_{eff} is ~230 nm. (**d**) PEEM images for square slits excited with right-handed circular and linear polarizations at 410 nm, the extracted effective wavelength λ_{eff} is also ~230 nm. The dot lattices can be observed for square slits excited with right-handed circular polarization, which support optical meron-like spin textures.

Supplementary Note 4
Two-photon photoemission process

For the excitation wavelength around 410 nm, two-photon photoemission process satisfies the condition of overcoming the work function of hBN, that is $P_E \propto I^2 \propto |\mathbf{E}|^4$, where P_E is the photoemission intensity, I is the local field intensity and $|\mathbf{E}|$ is the electric field amplitude. As shown in Supplementary Figure 7, the nonlinear order of approximately 2.0 is extracted from the power-dependent measurements from 10 to 220 mW for a ring slit at 410 nm with right-handed

circular polarization.

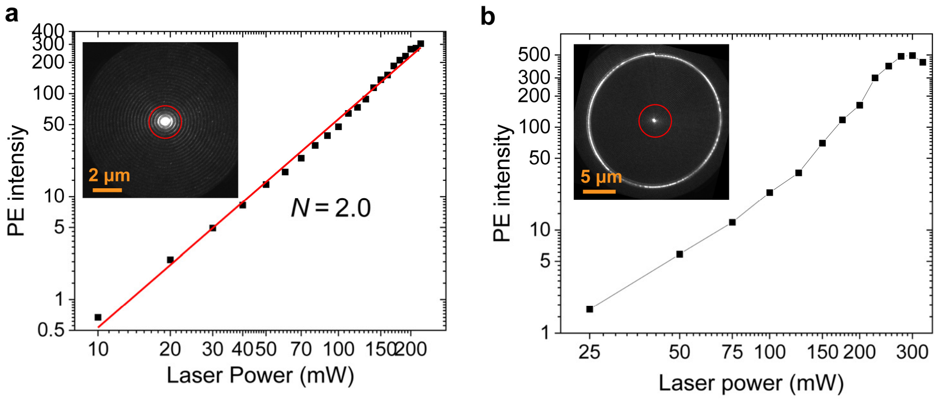

Supplementary Figure 7. Photoemission intensity versus laser power. (**a**) For the sample of $m = 0$ in Fig. 4 of the main manuscript, the nonlinear order $N = 2.0$ indicates a two-photon process. (**b**) For another sample with $m = 1$, at high laser power, the saturation and the following decrease of photoemission intensity with the increase of laser power is observed, which can be attributed to the slight surface charging at high laser power.

Supplementary Note 5

Discussions on the choice of conductive layer ITO and charging effect

It should be noted, a very thin ITO down to 10 nm is adopted in this work as conductive layer in order to avoid introducing absorption loss. ITO has much lower absorption loss than thin metallic film, such as gold and monolayer graphene. However, ITO itself still has a small absorption, therefore, the thickness down to 10 nm is preferred to maintain the low-loss hBN waveguide modes. The simulations for the influence of ITO thickness on the intensity of hBN waveguide mode are shown in Supplementary Figure 8.

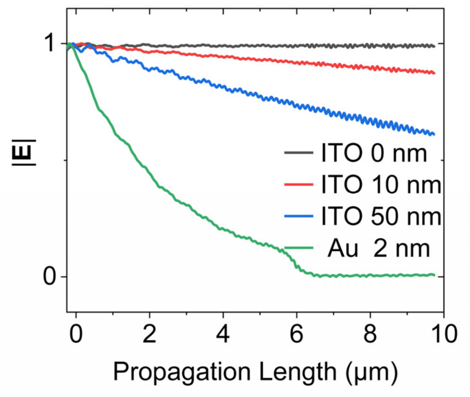

Supplementary Figure 8. Influence of ITO thickness on the intensity of hBN waveguide mode. Simulated decay of waveguide mode in hBN along propagation direction with different ITO layer thicknesses: 0 nm, 10 nm and 50 nm. The hBN waveguide with 2 nm Au as conductive layer is also plotted for comparison. The waveguide mode is excited from a line slit on 60 nm hBN with 410 nm laser at normal incidence.

The low loss of dielectric material is a prerequisite for strong photoemission enhancement. The enhancement is achieved by focusing the waveguide mode excited from the ring slit edge. That's to say, the waveguide mode is coupled into the hBN slab from the slit edge and propagates toward the center to form a strong nanofocusing. Therefore, the loss during propagation will have a vital influence on the final focusing intensity. From the simulations, without considering loss, the focusing intensity increases with the ring diameter, because more light is collected by the slit edge with a larger circumference. And the focusing intensity can be further largely enhanced by using circular grating coupler. As for SPP supported by metal, large loss is expected, resulting in weak enhancement. And it's expected that the focusing intensity cannot be efficiently enhanced by increasing the diameter due to the propagation loss of SPP. As shown in Supplementary Figure 9, by using the same design, the intensity of focusing spot with hBN waveguide is much larger than that with gold film. The intensity $|\mathbf{E}|^2$ at vacuum/hBN is ~12 times larger than that at vacuum/Au. In addition, the maximum intensity of hBN waveguide is not at the surface, but inside hBN, as shown in Supplementary Figure 9a, which is ~23 times larger than that at vacuum/Au. It should be noted, for SPP, the ring slit (m=0) excited with LCP does not form a focusing point in the center, but a doughnut pattern. To form a focusing point, spiral slit (m=−1) should be adopted and we use the focusing point to compare the intensity.

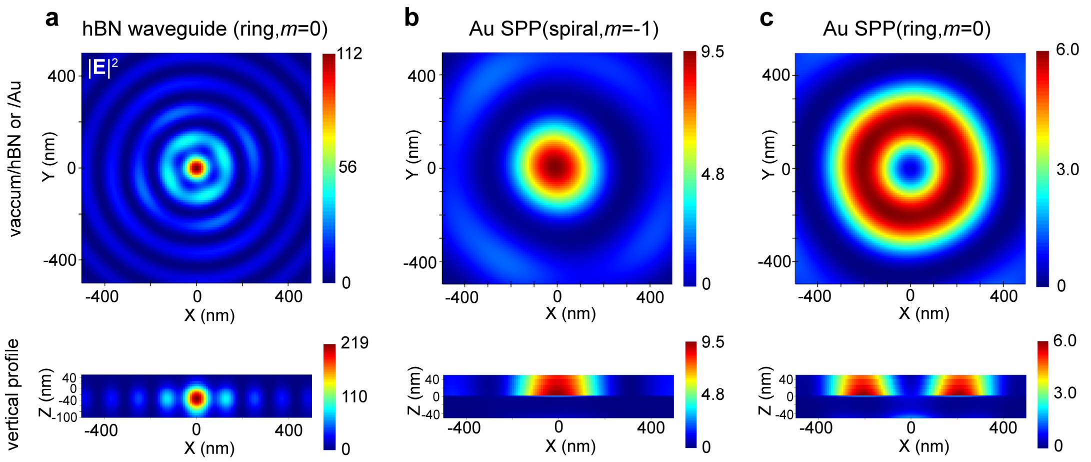

Supplementary Figure 9. Comparison of the nanofocusing intensities with hBN waveguide and SPP. (**a**) Intensity $|\mathbf{E}|^2$ for hBN waveguide excited with 410 nm LCP laser on a ring slit (radius 7.36 μm ($32\lambda_{\text{eff}}$), slit width 180 nm, hBN thickness 60 nm). (**b**) Intensity $|\mathbf{E}|^2$ for Au SPP excited with 800 nm LCP laser on a spiral slit (m=−1) (initial radius 7.02 μm ($9\lambda_{\text{eff\_spp}}$), slit width 200 nm, Au thickness 60 nm). (c) Intensity $|\mathbf{E}|^2$ for Au SPP excited with 800 nm LCP laser on a ring slit (radius 7.02 μm ($9\lambda_{\text{eff}}$), slit width 200 nm, Au thickness 60 nm).

In addition, we can try to measure the loss from the decrease of photoemission intensity during propagation. As shown in Supplementary Figure 10a,b, we measured the photoemission intensity along the propagation direction excited from a slit with 410 nm laser at normal incidence. The decay of photoemission intensity along the propagation direction can be observed. Considering the two-photon photoemission process, $P_E \propto I^2 \propto |\mathbf{E}|^4$, the electric field $|\mathbf{E}|$ along the propagation direction is extracted in Supplementary Figure 10c. From X = 2 μm to X = 7 μm, the amplitude of $|\mathbf{E}|$ (between the red dashed lines) decreases from 0.97 to 0.77, which means the amplitude of $|\mathbf{E}|$ has a 20% decrease in 5 μm. In contrast, in simulations, the amplitude of $|\mathbf{E}|$ has a 7.5% decrease in 5 μm. The larger loss measured in experiments could be due to fabrication quality and the inhomogeneous laser spot. Because the relatively small laser spot (~150 μm) was used in normal incidence, it's hard to evaluate the loss accurately, and the measured value should be larger than actual loss.

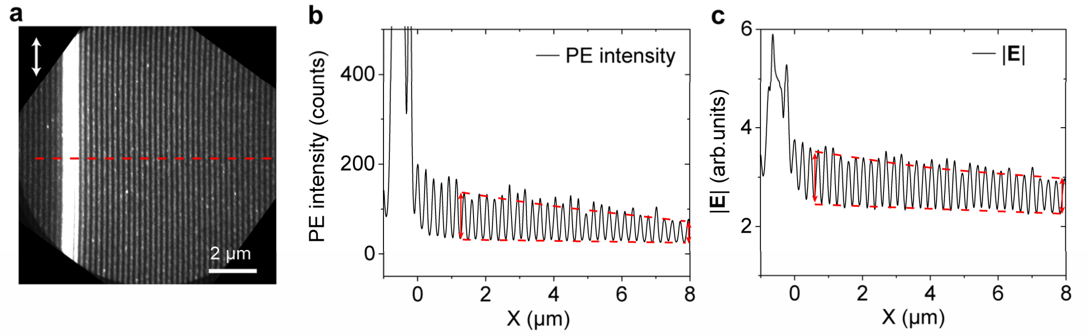

Supplementary Figure 10. Evaluation of propagation loss. (**a**) PEEM image for line slit excited with TE polarization with 410 nm laser at normal incidence. (**b**) crosscut line from (a). (**c**) Extracted amplitude of $|\mathbf{E}|$ following $P_E \propto I^2 \propto |\mathbf{E}|^4$, the amplitude of $|\mathbf{E}|$ has a 20% decrease in 5 μm.

The electron mean free path of hBN is on the order of 1~2 nm or less. The photoemission on hBN is directly from the surface layer (within 2 nm) of hBN, not from the ITO under the hBN, which means the electron mean free path has no direct connection with the thickness of hBN. For the charging effect, the hBN thickness up to 80 nm has no obvious charging phenomenon in our

study, because charges can be conducted through the underling ITO layer. In contrast, if the hBN and underling ITO are etched with a deep ring slit to be isolated from the surrounding ITO substrate, charging effect will appear easily, which means the ITO layer is very important in avoiding charging.

In addition, as shown in Supplementary Figure 7b, the excitation density could have some influence on charging effect, because we observed that when the laser power was above 300 mW, the photoemission intensity became saturated. This saturation effect could be attributed to the limited charge transfer efficiency from hBN to ITO.

To note, the hBN used in this study is relatively small flakes (in-plane size <300 μm), rather than film that coving the whole surface, which could also be an advantage for transferring charges from hBN to underlying ITO. In addition, the hBN flake can be clearly imaged by PEEM without any blur. For example, the hBN flakes can also be clearly imaged by PEEM with Si substrate.

The careful selection of materials to balance the bandgap and surface charging is needed because bandgap and conductivity is contradictory to some extent, large bandgap generally means low conductivity. To construct optical devices in visible range, large bandgap is preferred. However, common optical materials such as Si_3N_4, $LiNbO_3$ are not conductive, thus cannot be measured in PEEM. The materials with smaller bandgap such as Si, GaAs have sufficient conductivity, but they are not suitable for visible range due to large optical absorption. Therefore, the selection of materials is an important task. It could be a good idea to seek among novel materials. The van der Waals materials could be potential choices as they have special layered structure. And through the PEEM experiments, we found hBN is compatible with PEEM. It should be noted, it's hard to predict if the materials are suitable for PEEM measurements, the experimental attempts should be required.

Supplementary Note 6

PEEM images for near-field modes excited with right-handed circular and linear polarizations

For the Archimedean spiral slits with a series of geometrical charges ($+m$), the near-field vortex modes with right-handed circular polarization (SAM = −1) and linear polarization are shown in Supplementary Figure 11.

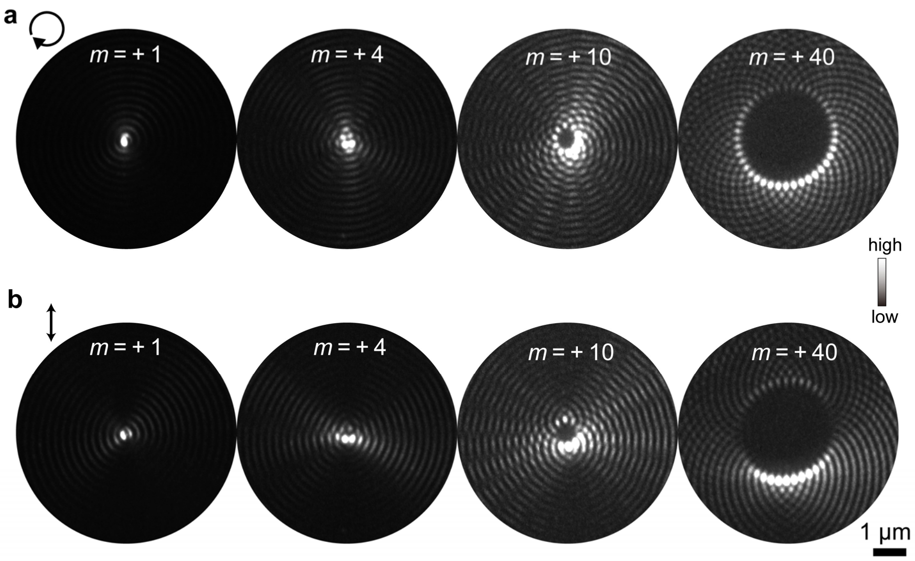

Supplementary Figure 11. PEEM images excited with other polarizations. PEEM images for near-field modes excited with right-handed circular (**a**) and linear polarizations (**b**).

Supplementary Note 7

<u>Discussion on the optical spin textures carried by vector near-field vortex modes</u>

The vector near-field vortex mode excited with a left-handed circularly polarized plane beam (SAM = +1, (E_x = 1, E_y = i)) on a ring slit (m = 0) is shown in Supplementary Figure 12, and the radius of the slit is set as $r_0 = 30\lambda_{\text{eff}}$.

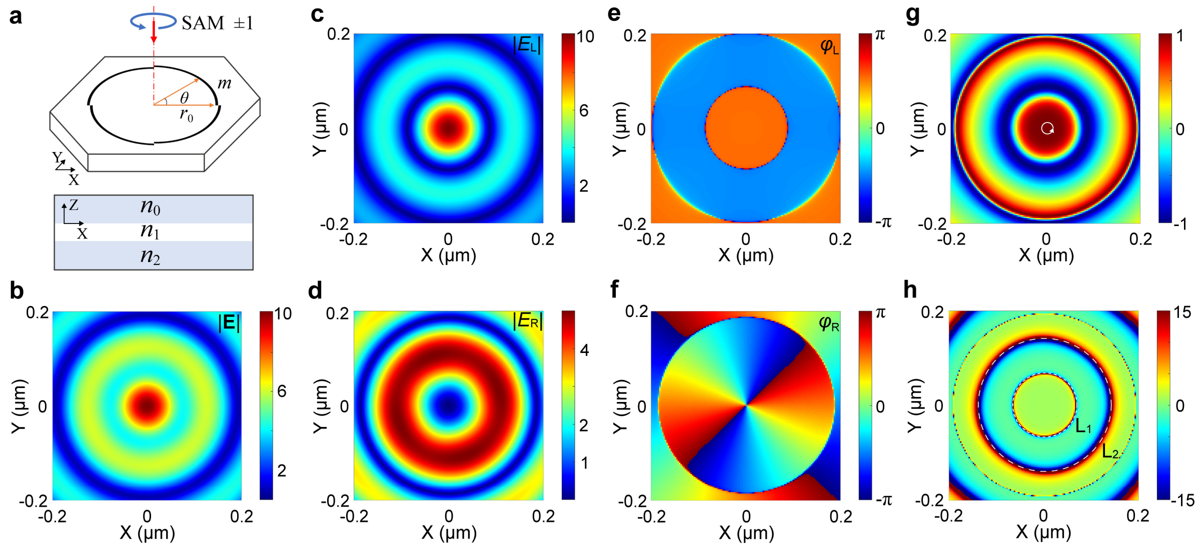

Supplementary Figure 12. Vector near-field vortex modes. (**a**) Designed structure of dielectric waveguide for the generation of near-field vortex, excited by free-space beam carrying spin angular moment

(SAM, ±1), with the etched ring or Archimedean spiral slits as couplers. the top and bottom claddings and the core of the waveguide are chosen as vacuum ($n_0 = 1$), glass ($n_2 = 1.5$), and hBN ($n_1 > n_2$). m is the geometrical charge introduced by Archimedean spiral slits. (**b**) Electric field (|**E**|) profile of the vector near-field vortex at the vacuum/hBN interface (top surface, Z = 0), excited with left-handed circularly polarized plane beam (SAM = +1) on a ring slit (m = 0, $r_0 = 30\lambda_{\mathrm{eff}}$). The decomposed electric field profiles of the vector near-field vortex by left- and right-handed circular polarizations (LCP/RCP) for amplitude (**c,d**) and phase (**e,f**). (**g,h**) Intensity ratios $(|E_{\mathrm{L}}|^2 \mp |E_{\mathrm{R}}|^2)/(|E_{\mathrm{L}}|^2 \pm |E_{\mathrm{R}}|^2)$ of the LCP/RCP components for the visualization of spin distribution, L_1 and L_2 in (h) denote the L lines.

For the top surface, that is the vacuum/hBN interface, the strongly focused electric field |**E**| profile is shown in Supplementary Figure 12b. The vector near-field vortex is decomposed into the scalar vortex with LCP/RCP as the bases, the corresponding amplitude and phase are shown in Supplementary Figure 12c–f, and the topological charge with $(m-1) = 0$ and $(m+1) = 2$ can be clearly observed from the phase variation. The intensity ratio $(|E_{\mathrm{L}}|^2 - |E_{\mathrm{R}}|^2)/(|E_{\mathrm{L}}|^2 + |E_{\mathrm{R}}|^2)$ of LCP/RCP components is displayed in Supplementary Figure 12g, with local left-handed circular polarization in the center of vortex and right-handed circular polarization at the periphery enclosed by the L-line (L_2) as noted in Supplementary Figure 12h through $(|E_{\mathrm{L}}|^2 + |E_{\mathrm{R}}|^2)/(|E_{\mathrm{L}}|^2 - |E_{\mathrm{R}}|^2)$, which implies the transition of SAM from "up" state in the center of vortex to "down" state at the periphery. The cycle-averaged Poynting vector $\mathbf{P} = \mathrm{Re}(\mathbf{E}^* \times \mathbf{H})/2$ is presented in Supplementary Figure 13a,c, where **E** and **H** are the electric and magnetic fields, indicating an in-plane rotating energy flux.

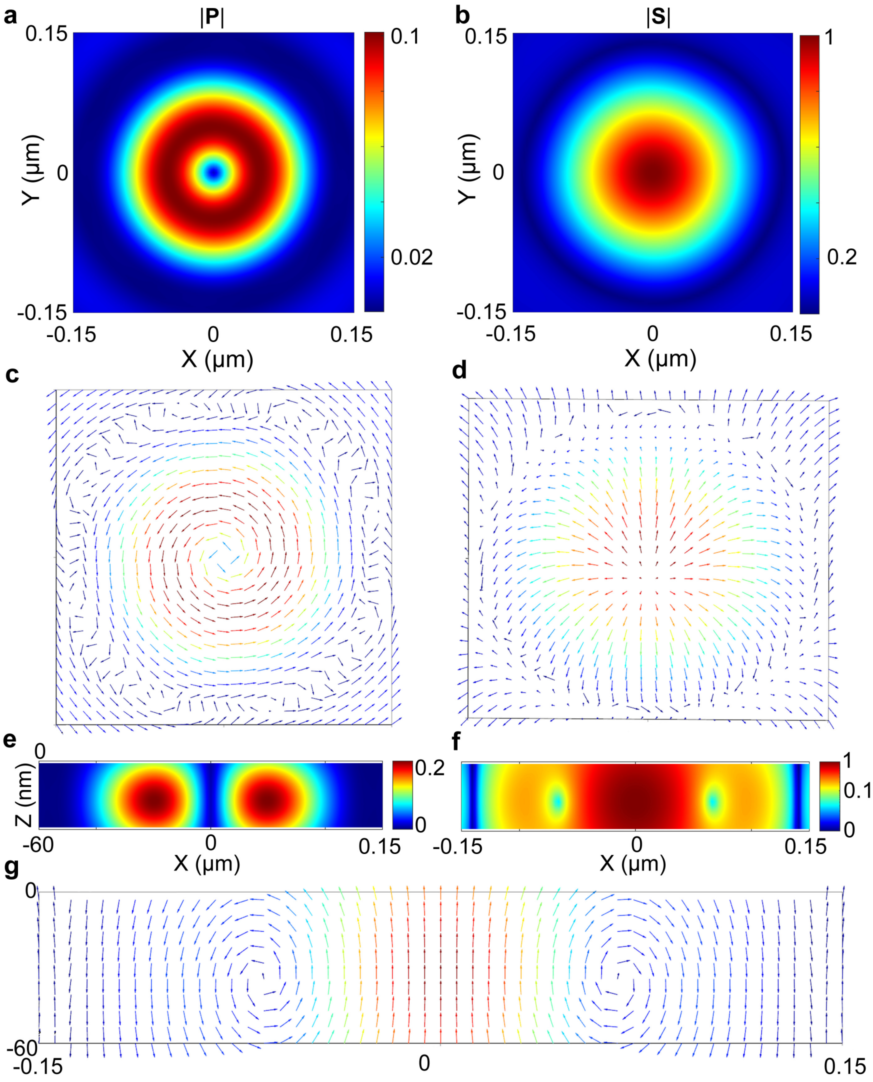

Supplementary Figure 13. Optical spin texture carried by vector near-field vortex modes. Amplitude profile (**a**) and vector mapping (**c**) of the Poynting vector for the near-field vortex in Supplementary Figure 12b at the top surface of the waveguide. (**b,d**) Corresponding amplitude profile and vector mapping of the SAM texture, respectively. (**e,f**) Vertical crosscut profiles (X-Z plane, Y = 0) of the Poynting vector and SAM texture, respectively. The color bar in (f) is in the log scale. (**g**) Vector mapping of SAM texture in the X-Z plane (Y = 0), showing the 3D toroidal-like spin texture. The intensity in (b) and (f) is normalized by the maximum in each image. The colors in vector mapping indicate the value of |**P**| or |**S**|.

To better analyze the SAM texture of the near-field vortex, the SAM of the vortex is calculated via[1–3]

$$\mathbf{S} = \mathrm{Im}(\varepsilon\mathbf{E}^* \times \mathbf{E} + \mu\mathbf{H}^* \times \mathbf{H})/4\omega \quad (1)$$

where, ω is the angular frequency, ε and μ are the permittivity and permeability, and the asterisks

denote complex conjugation. The SAM vectors at the top surface of hBN present a distinct Néel-type skyrmion-like spin texture (Supplementary Figure 13b,d), which is similar to that found in SPP vortex. From the center of the vortex to the periphery at the L-line (L_2), the SAM vectors rotate continuously from upward to the radial, and finally to downward direction. The topological charge N of the SAM texture is calculated by integrating the charge density within the periphery[2,3].

$$N = \frac{1}{4\pi} \iint \mathbf{S}_n \cdot \left(\frac{\partial \mathbf{S}_n}{\partial x} \times \frac{\partial \mathbf{S}_n}{\partial y} \right) dxdy \quad (2)$$

Where, $\mathbf{S}_n = \mathbf{S}/|\mathbf{S}|$ is the unit spin vector. The obtained N is close to 1, confirming the skyrmion topological character.

Distinguished from the SPP evanescent field at the interface, the Poynting vector is confined in the volume of the dielectric waveguide (Supplementary Figure 13a,e), manifesting the doughnut-shaped energy flux, with its maximum at the central plane of the waveguide, as seen from the vertical cross section. Considering the relationship of SAM and Poynting vector $\mathbf{S} \propto \frac{1}{2\omega^2} \nabla \times \mathbf{P}$ [2], the SAM texture also has a volume distribution (Supplementary Figure 13b,f). From the top surface to the bottom surface, the in-plane components of SAM gradually transfer from the positive radial, to azimuthal, and finally to the negative radial direction (Supplementary Figure 13g). This indicates that the SAM vectors transfer continuously from Néel-type at the top surface to twisted Néel-type when moving to the central plane, and then to Bloch-type near the central plane, and finally to Néel-type skyrmion texture with the opposite chiral whirl at the bottom surface.

We further investigate the spin textures carried by near-field vortices with high-order topological charges. In Supplementary Figure 14, the near-field vortex mode is excited with a left-handed circularly polarized plane beam (SAM = +1) on Archimedean spiral slits (m = 10). Similar results can also be obtained with a left-handed circularly polarized vortex beam (SAM = +1; OAM= +10) on a ring slit (m = 0). The ring-like spatial distribution of the Poynting vector is shown in Supplementary Figure 14a,c, which has a much larger size compared with that in Supplementary Figure 13a. At the top surface, the SAM vectors rotate continuously from upward to radial and finally to downward direction, surrounding the Poynting vector, which looks like a hollow skyrmion texture (Supplementary Figure 14b). As shown in Supplementary Figure 14d,e, the 3D SAM texture possesses a toroidal-like distribution surrounding the ring of the Poynting vector. The radius (R_s) of the Poynting ring or SAM toroid (from the vortex center to the maximum of the Poynting vector) increases nearly linearly with the topological charge of the vortex

(Supplementary Figure 14f), and R_s increases from 50 nm at $m = 0$ to 460 nm at $m = 10$. Therefore, the size of the Poynting ring or SAM toroid can be tuned by the topological charge of the near-field vortex. It should be noted, the discussions above are in an optical system with axial symmetry, where the 2D skyrmion-like texture supported by SPP is extended to 3D toroidal-like texture supported by dielectric waveguide. Following this idea, the skyrmion texture in hexagonal lattice and meron texture in square lattice supported by SPP can also be extended to corresponding 3D SAM texture in dielectric waveguide.

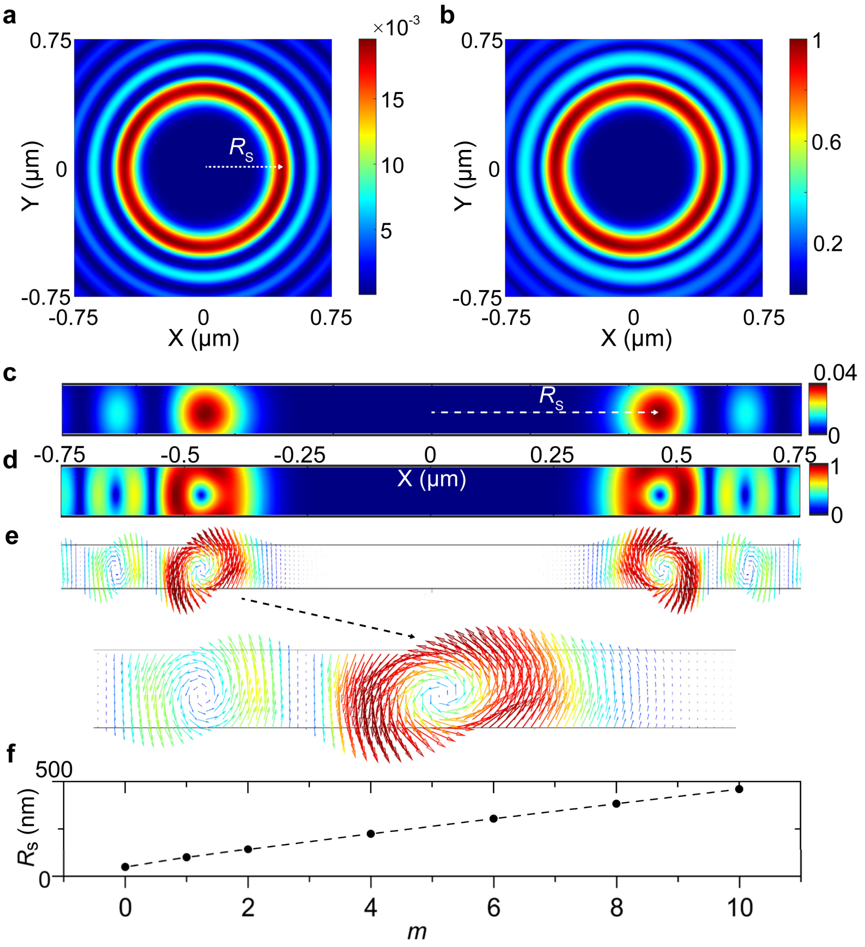

Supplementary Figure 14. Toroidal-like SAM texture. Toroidal-like SAM texture in near-field vortex with high order topological charge, excited with a left-handed circularly polarized plane beam (SAM = +1) on Archimedean spiral slits (m = 10, $r_0 = 30\lambda_{\mathrm{eff}}$). (**a,b**) Poynting vector and SAM texture at the top surface of the waveguide. (**d**) Poynting vector and (**d,e**) SAM texture in the X-Z plane. R_s denotes the radius of the Poynting vector or the toroidal-like spin texture. (**f**) R_s versus m.

The difference of TE mode supported by hBN waveguide and TM mode supported by SPP should be discussed more detailly. Firstly, as we all know, the electric and magnetic field components are correlated with each other in an electromagnetic field. The magnetic field component in the fundamental TE mode is similar or corresponding to the electric field component in the TM mode. That is to say, the magnetic field component in TE mode of hBN waveguide will have similar distribution with the electric field component of SPP. Considering this correlation, the cycle-averaged Poynting vector $\mathbf{P} = \mathrm{Re}(\mathbf{E}^* \times \mathbf{H})/2$ of TE mode of hBN will be similar to that of TM mode of SPP, and the SAM has an inherent correlation with Poynting vector. That is why TE mode supported by hBN waveguide at an interface has the similar SAM texture as TM mode supported by SPP.

Secondly, from the point of vector electromagnetic fields, taking the lowest vortex as an example, the TE mode is selectively excited from the ring slit by circularly polarized light, resulting in a phase retardation, similarly as that in TM mode of SPP. The phase retardation causes the formation of near-field vortex, and the SAM texture is in fact the inherent characteristic of the vortex. The vortex is a vector near-field vortex and can be decomposed into the scalar vortex with LCP/RCP as the bases. As shown in Supplementary Figure 12, the total vector electric fields are decomposed to LCP/RCP components, the LCP component carries no vortex topological charge and forms a focusing spot in the center, while the RCP component carries a vortex topological charge of 2 and therefore forms a doughnut field distribution with a rotating phase. It should be noted, the LCP and RCP components have opposite spin orientation, therefore, the nesting of LCP/RCP components results in the spin-flipping structure, that is SAM texture.

Supplementary Note 8
Full-scale images for TR-PEEM measurements

The images in Fig. 3a of the main manuscript are cropped from the full-scale images as shown in Supplementary Figure 15.

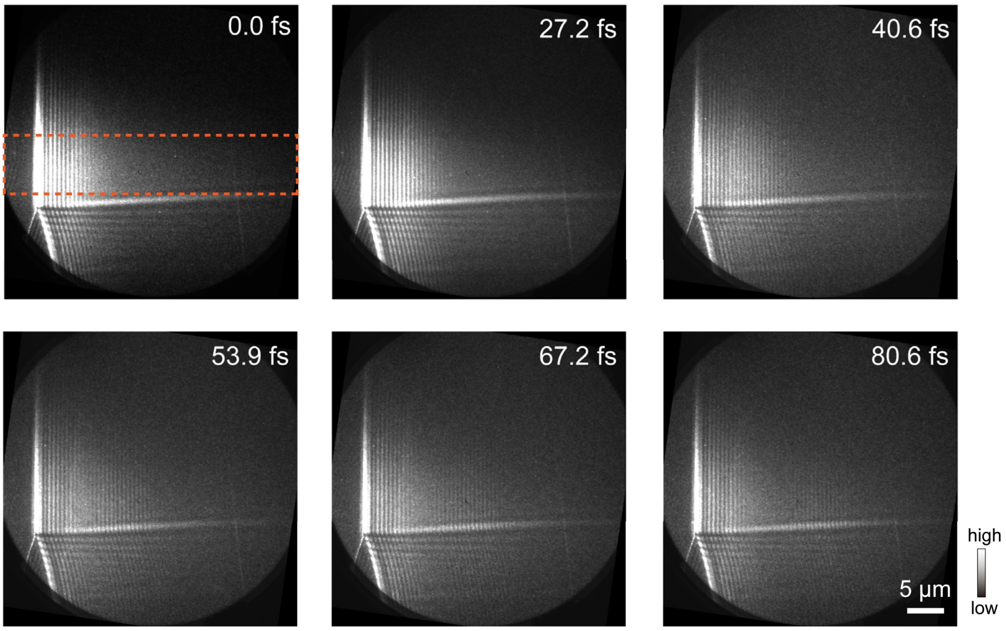

Supplementary Figure 15. Full-scale images. Full-scale images for TR-PEEM measurements in Fig. 3a of the main manuscript.

In addition, the near-field modes excited with ring slits at oblique incidence are presented in Supplementary Figure 16, where the excitation of waveguide modes from the left side of the ring slit and the following propagation and focusing are observed.

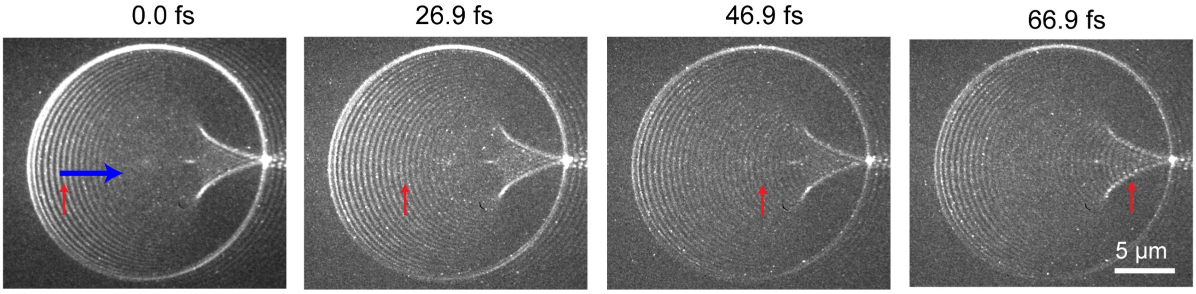

Supplementary Figure 16. TR-PEEM images for ring slit. TR-PEEM images for ring slit at several time delays, manifesting the excitation from the left edge of slit and the propagation and focusing to the right side.

Supplementary Note 9
<u>Calculations for group velocity</u>

The group velocity can be extracted from the moving of wave packet by the relationship:

$$v_g = \frac{c}{n_g} \tag{3}$$

$$n_g = \frac{c}{v_{observed}} + \sin\theta\cos\alpha \quad (4)$$

Where, $v_{observed}$ is the observed moving velocity of the wave packet in the normal direction vertical to the slit coupler. θ is the incidence angle of laser with the normal direction of sample plane and $k_0\sin\theta$ is the in-plane wave vector of the incident laser, α is the angle between in-plane wave vector $k_0\sin\theta$ and the normal direction of the slit coupler. Here, $v_{observed} = 2.4 \times 10^8$ m/s as extracted from Fig. 3d of the main manuscript, $\theta = 74°$, $\alpha = 8°$, the calculated $n_g = 2.2$. The derivation of this relationship can be found in references[4,5].

In addition, the simulation results for group velocity are shown in Supplementary Figure 17. The wave packet moves from position at 3 μm to 8 μm with 36.7 fs. Then, the group velocity is calculated to be 0.137 μm/fs, and the corresponding group refractive index $n_g = 2.2$, well consistent with the experimental result.

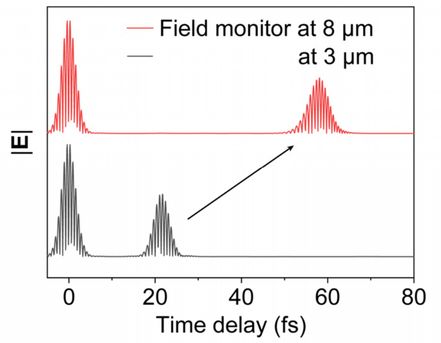

Supplementary Figure 17. Simulation results for group velocity. Simulated wave packet moving from position at 3 μm to 8 μm with 36.7 fs. The group velocity is calculated to be 0.137 μm/fs, and the corresponding group refractive index $n_g = 2.2$, well consistent with the experimental result.

Supplementary Note 10

Comparison of photoemission properties for near-field vortices with different topological charges

The photoemission properties for near-field vortex with different topological charges are the same in momentum and energy spaces, due to the atomically flat surface of hBN, as shown in Supplementary Figure 18.

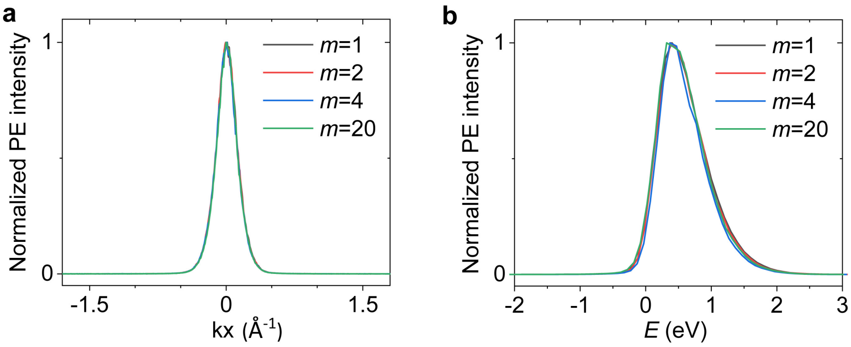

Supplementary Figure 18. Photoemission properties for near-field vortices with different topological charges. Comparison of photoemission properties for near-field vortices with different topological charges in momentum (**a**) and energy spaces (**b**) excited with 410-nm laser with right-handed circular polarization at normal incidence.

Phenomenally, the photoemission of hBN with 410 nm laser is a two-photon process, as shown in the power-dependent measurements, i.e., two photons (2×3.02 eV) are absorbed to overcome the work function of hBN. Considering the complicated band structure of hBN, the underling physics of photoemission should be discussed, including the contributions of secondary electrons, intervalley scattering and interband defect states. It has been reported that photoemission signals are dominantly from secondary electrons in the low kinetic energies for metal, in particular for energies close to Fermi level[6]. For wide-bandgap hBN, secondary electrons could also have a contribution to the low energies of photoemission spectrum, but the contribution could be less important than that in metal due to the large bandgap of hBN. In addition, photoexcitation should occur in multiple valleys of hBN band structure, including the valleys along L-M direction[7–9]. In our experiment, only the photoelectrons around the Γ point are observed, simply due to the small photoemission horizon set by the limited photon energies. The intervalley scattering from other valleys toward Γ point could also have a contribution to the final photoemission signals. What's more, defects in hBN could be possibly introduced by sample preparation and laser illumination, the defect states in the wide bandgap could also assist the two-photon process by creating actual intermediate energy levels. Therefore, multiple effects could have contributions to the photoemission process. However, it's hard to evaluate how important of each effect in current stage and is out of the main claims of the manuscript. More detailed investigations on the photoemission mechanism of hBN could be performed in the future.

Supplementary References

1. Aiello, A., Banzer, P., Neugebauer, M., Leuchs, G. From transverse angular momentum to photonic wheels. *Nat. Photonics* **9**, 789–795 (2015).
2. Du, L., Yang, A., Zayats, A.V., Yuan, X. Deep-subwavelength features of photonic skyrmions in a confined electromagnetic field with orbital angular momentum. *Nat. Phys.* **15**, 650–654 (2019).
3. Dai, Y. et al. Ultrafast microscopy of a twisted plasmonic spin skyrmion. *Appl. Phys. Rev.* **9**, 011420 (2022).
4. Lemke, C. et al. Mapping surface plasmon polariton propagation via counter-propagating light pulses. *Opt. Express* **20**, 12877–12884 (2012).
5. Hartelt, M. et al. Energy and Momentum Distribution of Surface Plasmon-Induced Hot Carriers Isolated via Spatiotemporal Separation. *ACS Nano* **15**, 19559–19569 (2021).
6. Knoesel, E., Hotzel, A., Hertel, T., Wolf, M. and Ertl, G. Dynamics of photoexcited electrons in metals studied with time-resolved two-photon photoemission. *Surf. Sci.* **368**, 76–81 (1996).
7. Blase, X., Rubio, A., Louie, S.G. and Cohen, M.L. Quasiparticle band structure of bulk hexagonal boron nitride and related systems. *Phys. Rev. B* **51**, 6868 (1995).
8. Hunt, R.J., Monserrat, B., Zólyomi, V. and Drummond, N.D. Diffusion quantum Monte Carlo and G W study of the electronic properties of monolayer and bulk hexagonal boron nitride. *Phys. Rev. B* **101**, 205115 (2020).
9. Artús, L. et al. Ellipsometry Study of Hexagonal Boron Nitride Using Synchrotron Radiation: Transparency Window in the Far-UVC. *Adv. Photonics Res.* **2**, 2000101 (2021).
